# Can Predation Pressure Help Explain the Curious Evolution of Ballistic Seed Dispersal?

**DOI:** 10.1002/ece3.71081

**Published:** 2025-03-13

**Authors:** C. R. Sharpe, G. D. Ruxton

**Affiliations:** ^1^ Conservation Ecology Group, Department of Biosciences Durham University Durham UK; ^2^ School of Biology, Centre for Biological Diversity, Institute of Behavioural and Neural Sciences University of St. Andrews St. Andrews UK

**Keywords:** autochory, ballochory, predation pressure

## Abstract

Ballistic seed dispersal (ballochory) involves the autonomous explosive release of seeds from adult plants. The unconventional mechanics of this strategy have understandably drawn considerable scientific attention. The explosive release of seeds is achieved by a variety of physical mechanisms but broadly involves the rapid coiling or shattering of seed pods to transfer kinetic energy to seeds, facilitated largely by either the evaporation or absorption of water in seed pod tissues. There has been a bias toward researching physiological and physical aspects of ballistic plants, with the evolutionary ecology being comparatively neglected. Although ballochory is represented in 23 plant families, it has never become common. This fact should invite curiosity regarding the selective pressures that encourage its evolution. Previous research has been unable to correlate ballochory with plant traits such as morphology, generation time or habitat preferences, and so we take an alternative approach in considering the evolutionary advantages that can provide insight on the shared set of circumstances that favour the evolution of this strategy. We review the known selective advantages that ballistic dispersal can confer to plants and promote a hypothesis that ballochory may be particularly selected for in instances of concentrated predation pressure on parental canopies. For plants in static and patchy landscapes, such a strategy could balance a trade‐off between escaping concentrated natural enemies while maximising the probability of transport to suitable habitat. We account for its rarity by considering the major opportunity cost that may only be justified when other seed dispersal mechanisms are limited. Moving forward, we suggest experimental manipulations to test this hypothesis and promote a research agenda in the field of ballistic seed dispersal that illuminates its intriguing evolution.

## Abstract 2

This viewpoint article describes an initial hypothesis regarding the evolutionary ecology of explosive seed dispersal. Although the physical mechanisms of this unconventional process are well understood, the evolution of this plant trait has received comparatively little attention. We suggest that predation pressure on or beneath the parent plant could help explain the repeated evolution of this trait and hope to stimulate experimental manipulations to test this.

## 1 Introduction

While most plants rely on external agents to transport their seeds, self‐powered seed dispersal (autochory) offers an alternative set of dispersal benefits to plants. Autonomous control of seed dispersal allows plants to avoid dispersal limitations encountered through relying on external vectors. For instance, plants of species that rely on frugivores for dispersal services can experience no seed dispersal if appropriate animal vectors are not present (Carlo and Morales 2008); whereas plants using ballistic dispersal can reliably autonomously disperse all of their seeds. Autonomous seed dispersal strategies are more common at higher latitudes and elevations, and in savannas, grasslands, deserts, and alpine environments (Rogers et al. 2021), although the occurrence of ballochory in a wide range of habitats has confounded efforts to offer a simple generalisation on the circumstances where it occurs.

Ballistic seed dispersal or ballochory permits greater dispersal distances than alternative autochorous strategies (Table 1). This is achieved by the explosive release of seeds from adult plants. Although different mechanisms are used to achieve this, the process generally involves dehiscence of multiple seed‐containing valves or capsules that constitute a fruit or seed pod. Either by absorbing or evaporating water, the forces required to initiate explosive dehiscence are generated within the plant (for review see: Sakes et al. (2016)).

**TABLE 1:** Summary of forms of self‐powered seed dispersal.

Method of autochory	Description	Typical dispersal distance
Barochory	Plants drop seeds to the ground, dispersal by gravity. Often followed by dispersal by animals (Tella et al. 2019)	< 1 m
Herpochory	Self‐propelling by structural alterations during successive wet and dry conditions that allow seeds to ‘crawl’ across the substrate (Stamp 1989)	< 1 m
Blastochory	Sprawling plant stems carry seed away from parent plant, frequently involving secondary movement by an external vector (Vittoz and Engler 2007)	< 1 m
Ballochory	Explosive release of seeds after dehiscence of seed pod (Hofhuis and Hay 2017)	< 5 m

Rather than being simply an oddity of natural history, explosive seed dispersal is widespread, occurring on all continents other than Antarctica. It can be locally common; approximately 8% of New Zealand plant species exhibit ballochory (Thorsen et al. 2009). Perhaps the historically reduced availability of animal seed dispersers in New Zealand (Thorsen et al. 2009) has provided a selection pressure for self‐dispersal. By the same logic, it has been suggested that ballochory could have been more commonly relied upon by plants prior to the diversification of mammals and birds in the late Cretaceous and the plant adaptations for animal dispersal that accompanied this (Roberts and Haynes 1983). Ballochory has evolved multiple times, represented in 23 plant families including *Fabaceae* (Neubert and Parker 2004), *Euphorbiaceae* (Rickert and Fracchia 2010), *Geraniaceae* (Stamp 1989), *Polemoniaceae* (Stamp and Lucas 1983) and *Oxalidaceae* (Rezvani et al. 2010). Ballistic species can also be widespread, demonstrated by the pervasive *Impatiens glandulifera*, Himalayan balsam, which has invaded nearly all of Europe (Pysek and Prach 1995) and the potential for rapid expansion and colonisation by plants using this dispersal mode has equally been exhibited by hairy bittercress, *Cardamine hirsuta* , with its establishment on five continents outside of its native range (Lihová et al. 2006; Matsuhashi et al. 2016).

The inherently dynamic physical processes that underlie explosive seed dispersal have been well researched (Hayashi et al. 2009, 2010; Evangelista et al. 2011; Vaughn et al. 2011; Deegan 2012; Sakes et al. 2016; Poppinga et al. 2019; Li et al. 2020; Neumann and Hay 2020; Jorge and Patek 2023; Hesse‐Withbroe and Whitaker 2024). While this has provided fascinating insight into an eye‐catching phenomenon, equally interesting questions in the realm of evolutionary ecology have been overshadowed up to now. The evolutionary ecology of several similarly uncommon forms of seed dispersal (dispersal by fish, reptiles or external transport by animals) has been well considered in contemporary reviews (Sorensen 1986; Correa et al. 2007, 2015; Valido and Olesen 2007), while ballochory has experienced less consideration despite sustained research since the 1970s.

Previous comparative analyses have been unable to identify a strong, consistent relationship between any life‐history or ecological trait and the adoption of ballochory (Stamp and Lucas 1983; Thomson et al. 2010). This established lack of an observed relationship between ballistic species and plant traits has motivated us to consider an alternative line of inquiry in this manuscript. Setting aside differences in morphology, life‐history and ecological traits among ballistic species, we instead collate existing evidence regarding the potential suite of selective benefits and costs of ballistic dispersal. We use this evidence to reason that there is a set of circumstances that have promoted the repeated evolution of this strategy. While we promote the hypothesis that predation pressure can provide the selective impetus for ballistic dispersal, we also consider how escaping competition or achieving directed dispersal could (alternatively or additionally) drive selection for this plant trait. To account for the rarity of ballochory, we suggest that major opportunity costs only justify ballochory in environments where alternative dispersal mechanisms are limited.

## 2 The Evolutionary Ecology of Seed Dispersal

For plants, seed dispersal represents the only mobile stage in their life history, and there are several advantages available to plants by transporting their seeds. Dispersal can mitigate kin competition (Cheplick 1992), facilitate range expansion by colonising new habitats (Kaproth et al. 2023), remove seeds from areas of concentrated resources where predation is high (Janzen 1970; Connell 1971), or result in the transport of seeds to favourable sites for germination and establishment (Green et al. 2009). When any of these is achieved by dispersal, seeds that have been dispersed can have higher chances of survival and reproduction than undispersed seeds (Howe and Miriti 2004). These selective forces can be broadly grouped into the escape, colonisation, and directed dispersal hypotheses to explain plant adaptation for dispersal.

Often, seed mortality is elevated near maternal plants due to concentrated neighbourhoods of natural enemies. Firstly, it is reasonable to expect that seed predators are present at a higher density on or around adult plants due to the concentrated resource, but seed mortality can also be encountered through kin competition or infection by pathogens. Therefore, dispersal away from the parent plant allows seeds to ‘escape’ such concentrated natural enemies: the escape hypothesis (Janzen 1970; Connell 1971).

A further possible selective advantage of seed dispersal is the capacity to move seeds to new habitats. By dispersing seeds, plants improve the chances that seeds land in a favourable habitat for germination and recruitment (Howe and Smallwood 1982). In highly variable environments, wide dispersal can therefore be interpreted as a bet‐hedging strategy (Levin et al. 2003); by spatially distributing offspring, the odds that some will be successful increase. This is not only important for colonising distant sites, but seed dispersal to local vacant sites is crucial in ecological succession (Howe and Miriti 2004). Evidence supports this, and, even within one species, a population that exhibits wider dispersal can experience more rapid range expansion compared to a population with less dispersal (Kaproth et al. 2023). The success of many invasive plant species can be attributed to dispersal strategies; greater seed production in invasive species increases the probability of long‐distance dispersal and subsequent establishment relative to native species (Mason et al. 2008). Taking one example of an invasive wind‐dispersed plant, *Gladiolus gueinzii*, increased dispersal ability in plants at the expansion frontier facilitates faster spread (Tabassum and Leishman 2018). Plant invasions are thus driven by effective dispersal, and researchers strive to understand the genetic basis and phenotypic divergence that fuels successful invasions (Keller and Taylor 2008; Prentis et al. 2008).

Finally, the directed dispersal hypothesis proposes that adaptations for dispersal are selected for when these adaptations promote directed transport to non‐random locations that improve the chances of germination and survival (Howe and Smallwood 1982). Examples of empirical support for this hypothesis come from ant‐dispersed shrub species, where seeds flourish on nutrient‐rich ant mounds where they are deposited by ant dispersers (Davidson and Morton 1981), as well as the transport of mistletoe, *Plicosepalus acacia*, to host trees by yellow‐vented bulbuls, *Pycnonotus xanthopygos* (Green et al. 2009).

Although theories on the advantages of dispersal (escape, colonisation, directed dispersal) stress the advantages of moving seeds away from parent plants, there are cases where it is beneficial for plants to produce seeds with lower dispersal abilities and delayed germination that enable population persistence at the parent site, producing dimorphic seeds in certain species (Miguel et al. 2017).

## 3 Advantages of Ballistic Dispersal

Using ballistic dispersal, plants can generally move their seeds less than five metres (Stamp 1989; Stamp and Lucas 1990; Ohkawara and Higashi 1994; Narbona et al. 2005; Beaumont et al. 2009; Yoshikawa et al. 2018), although some exceptional canopy trees can launch their seeds several tens of metres (vander Burgt 1997; Norghauer and Newbery 2015). Along with dispersal by ants, ballistic dispersal therefore represents a relatively short‐range strategy compared to dispersal by wind, water or vertebrate animals (Thomson et al. 2010). This does not prevent the method from providing the benefits of seed dispersal discussed in the previous section, but such benefits are likely most relevant to relatively small plants, generally with canopy diameters less than 4 m (Hughes et al. 1994). Dispersal strategies are dependent on landscape features, and to optimise the chances of seeds encountering favourable habitat post‐dispersal, short dispersal distances are selected for in static and patchy landscapes (Treep et al. 2021). More connected populations facilitated by shorter dispersal distances also promote persistence and reduced extinction risk (Molofsky and Ferdy 2005). Here, we consider evidence from ballistic species to better understand the selective benefits of this strategy.

### 3.1 Escape From Predators and Competitors

Evidence from multiple species demonstrates that ballistic dispersal can remove seeds from concentrated natural enemies at parent plants. In *Euphorbia balsamifera*, plants can transport the entirety of the seed crop beyond the parental canopy with explosive dispersal (Berg 1990), but more commonly a more modest proportion of seeds are moved this critical distance: *Adriana quadripartita* moves 45% of its seeds beyond the shadow of the parental canopy by ballochory (Beaumont et al. 2009), and *Vicia sativa* plants ballistically disperse half of their seeds between 89% and 96% of the maximum possible distance allowed by the plants' mechanical apparatus (Garrison et al. 2000); evidencing selection for increasing the dispersal distance.

It is reasonable to question whether such short dispersal distances are functional in relation to escaping predators. High seed predation levels on the canopies of ballistic species have been documented in several circumstances: heavy pre‐dispersal predation by slugs and caterpillars on the unripe ovaries of *Viola* species (Beattie and Lyons 1975), high pre‐dispersal predation by weevils, wasps and caterpillars in an arid environment (Fischer et al. 2015), heavy damage from host‐specific herbivores noted for *Impatiens* species (Schemske 1978), the loss of up to 30% of seeds prior to ballistic dispersal in *Crotalaria podacarpa* (Fischer et al. 2015), and avian pre‐dispersal predation in the Japanese star anise *Illicium anisatum* (Yoshikawa et al. 2018). Since a high proportion of predators concerned are invertebrate herbivores such as slugs, weevils, and caterpillars (Yano 1997; Fischer et al. 2015), dispersing seeds only a few meters away from the parent effectively escape these relatively slow‐moving animals that are themselves vulnerable to predation on the ground (Ferrante et al. 2017). Even more mobile predators could have reason to concentrate their foraging underneath parental canopies that could provide shelter and safety from their own predators. For instance, one study suggests rodents are ten times more likely to consume seeds underneath plant canopies, even when seeds are present at higher density in the open (Odowd and Hay 1980).

Investment in defensive structures is related to a plant's risk of attack (Stamp 2003), so it is reasonable to infer that ballistic species with defensive adaptations experience high predation pressure on the plant. Ballistically dispersing species *Jatropha hieronymi* and *Jatropha excisa* both possess chemical defences that deter ant, arthropod and vertebrate seed predators (Rickert and Fracchia 2010), lending support to ballochory being an anti‐predator defence. Further, elevated predation pressure appears to drive a divergence between the purely ant‐dispersed syndrome of *Viola* and the diplochorous syndrome that uses ballochory prior to ant‐dispersal, implicating ballochory as a predator escape strategy (Beattie and Lyons 1975). *Viola* species that use ballistic dispersal have taller, thickened stalks beneath their seed pods compared to solely myrmecochorous species, as well as thicker, woody seed capsules; both adaptations that would appear to protect seed pods from pre‐dispersal predation. Compared to purely myrmecochorous species of *Viola* that rely on ant‐dispersers to escape predation near the parent plant, these authors posit that selection for diplochorous forms has been driven by circumstances where the conditions are not favourable for precise coevolution between ants and plants, and the dominant selective force has been predation.

Although it is theoretically feasible that ballochory is a predator escape strategy, there is a current lack of empirical evidence to suggest ballistic dispersal can provide a functional escape from larger or more mobile predators such as rodents, flying insects, larger arthropods or grazing herbivores. As such, we would promote this as a line of future enquiry. It would also be valuable for researchers to quantify the predation pressure experienced by ballochorous species relative to closely related non‐ballochorous species.

In addition to the dispersal distance offered by ballochory, it is useful to consider the adaptive benefits of the entire dispersal kernel (Treep et al. 2021). As well as the predator escape function of transporting seeds away from parent plants, by scattering seeds, the density of dispersed seedlings is reduced compared to non‐dispersed or passively dispersed seeds. Where other forms of autochory produce a clumped deposit of seeds, explosive release from plants propels seeds away from each other. Due to the negative effects of sibling competition on growth, survival, and reproduction (Cheplick 1992), the selective benefits of ballochory could therefore also lie in the reduction of competition between seedlings. Since dispersal determines sibling density, dispersal therefore influences its own evolution through density‐dependent processes (Wender et al. 2005).

### 3.2 Facilitating Directed Dispersal With Ballochory

As well as reducing negative density‐dependent mortality of seeds, the scattering of seeds following explosive release can also encourage the collection of seeds by secondary dispersers that provide directed dispersal (Ohkawara and Higashi 1994). In an experimental setting, Ohkawara and Higashi (1994) demonstrated that a greater proportion of seeds were removed and dispersed by ants when they were scattered as opposed to single clumped deposits. Secondary dispersal by ants (myrmecochory) is facilitated by a mutualism: seeds develop a nutrient‐rich fatty acid reward (the elaiosome) that is consumed by ants after carrying the seed some distance (Beattie and Lyons 1975; Stamp and Lucas 1990; Ohkawara and Higashi 1994; Beaumont et al. 2009; Rickert and Fracchia 2010). In Australia, where ballistic plants frequently display secondary myrmecochory, ants have been found to increase the proportion of *Adriana quadripartita* seeds that are transported beyond the parental canopy after ballistic dispersal from 45% to 77% (Beaumont et al. 2009). In *Cnidoscolus stimulosus* , *Crotalaria rotundifolia* , and *Stillingia sylvatica* , secondary myrmecochory augments initial ballistic dispersal distances by an average of 5.8 m to increase the proportion that move beyond the parental patch (Stamp and Lucas 1990).

While secondary myrmecochory can increase dispersal distance, the main selective advantage of transport by ants more likely lies in directed dispersal to sites where germination chances are improved. Heightened nitrogen and phosphorus at ant nests and refuse piles (Vander Wall and Longland 2004) improve the survival and germination chances of dispersed seeds (Giladi 2006; Traveset et al. 2014), to an extent that plants are in competition to recruit ant dispersers that will provide this transport (Davidson and Morton 1981). Despite aggregations at ant nests, evidence contradicts the notion that seeds experience negative density‐dependent effects, with seeds having increased reproductive output compared to seeds in surrounding soil despite higher conspecific densities (Rissing 1986). At ant nests, seeds may also benefit from protection from predators through ant defensive behaviours around nests (Heithaus 1981). Odowd and Hay (1980) observe that by moving *Datura discolor* seeds only 1–3 m away from the parental canopy, ants reduce the probability of predation from 30% to only 3.5%. This also supports our earlier discussion that even short‐range dispersal can be functional in relation to predator escape.

In the past, it was believed that a trade‐off must exist between maximising dispersal distances by ballochory and maximising secondary dispersal due to morphological constraints on seeds (Beattie and Lyons 1975; Stamp and Lucas 1983); however, subsequent research has established a positive correlation between investment in the two strategies (Rickert and Fracchia 2010; Chen et al. 2019). Therefore, using this diplochorous dispersal syndrome can bring combined benefits to plants, where primary ballochory could provide an escape from predators and competitors concentrated at parent plants, while secondary myrmecochory provides directed dispersal to advantageous sites for establishment. Ant‐dispersed plants release their seeds earlier in the year than plants relying on other dispersal agents, increasing their probability of dispersal (Oberrath and Böhning‐Gaese 2002), and we propose that ballistic dispersal could provide a means for control over this timing of seed release as a self‐powered strategy that maximises the chances of dispersal for seeds. Compared to alternative autochorous strategies, the scattered dispersal kernel increases the efficiency of secondary ant dispersal (Ohkawara and Higashi 1994).

Here, we have attempted to summarise evidence regarding the benefits of ballistic dispersal. From the previous section, we have emphasised that dispersed seeds have a higher per‐capita success compared to undispersed seeds (Terborgh et al. 2011) whether that be due to escape from predators, escape from competitors, colonising new areas or experiencing transport to non‐random locations where chances of success are improved. Among dispersal strategies, ballochory uniquely offers plants autonomy in their access to these benefits. Relying on external vectors to provide seed dispersal represents a risk to plants that dispersal will be limited by the chance appearance of appropriate vectors. In extreme environments where encounters with animal dispersers are less frequent, autonomous dispersal is a more common strategy, and in the context of contemporary defaunation, dependence on animal dispersers is ever more precarious (Rogers et al. 2021). Therefore, a major selective advantage of ballochory lies in the strategy's ability to free plants from the inherent uncertainty of external vectors. Compared to the other autochorous strategies that also offer reliable autonomous dispersal, ballochory's distinct ability to transport seeds beyond the shadow of their parental canopy translates to reduced negative impacts of density‐dependent predation and competition concentrated on parent plants, as well as increased recruitment of secondary ant dispersers. In line with existing evidence, we therefore propose that ballochory could be selected for in circumstances where a short dispersal distance optimises the chances of encountering suitable habitat and where plants experience selection pressure from aggregated predators or competitors beneath the parent plant. Next, we consider the features of ballochory that can account for its rarity.

## 4 Costs of Ballochory

Despite the repeated evolution of ballochory, it remains an uncommon strategy of seed dispersal among extant plants. We believe this can be explained by a complex of associated features. The evolution of seed dispersal traits in general is constrained by the required investment in physical dispersive structures (Bonte et al. 2012; Cullen and Hay 2024). Greater dispersal distances are possible when plants can afford more investment in their fruit or seed pods (Rickert and Fracchia 2010; Jacobs and Lesmeister 2012); therefore optimising ballistic dispersal requires allocating resources to fruit or seed pods (Kaproth et al. 2023). This represents an opportunity cost—the hypothetical expense of investing in ballochory rather than another mutually exclusive dispersal strategy (Bonte et al. 2012). Further, by adapting for ballochory, a set of constraints is imposed upon the size, weight, and shape of seeds to optimise dispersal by explosive release. The seeds of ballistic plants are rarely heavier than 100 mg (Hughes et al. 1994), and since larger seed size is functional in relation to environmental hazards (Westoby et al. 1996; Kidson and Westoby 2000), such a size limit could reduce the resources available to support seeds during carbon deficits, relative to larger seeds relying on alternative forms of dispersal. The adaptations that promote optimised ballistic dispersal do not align morphologically with adaptations for transport by long‐distance dispersal vectors: wind, water, or vertebrate animals. The ingestion of fruits by vertebrate frugivores, such as migrating passerines, can transport seeds several thousands of meters (Costa et al. 2014), allowing spreading from one isolated habitat to another. The same feat can be achieved by wind dispersal (Corlett 2009) and attachment to the outside of animals (Manzano and Malo 2006), but not by ballistic dispersal. Therefore, the morphological constraints on seed size for ballochory appear to represent a very high opportunity cost to the strategy that can perhaps account for its rarity among extant plants. Possibly, plants using this strategy are remnants of a time when alternative dispersal strategies were extremely limited, prior to the diversification of mammals and birds. As suggested by Roberts and Haynes (1983) ballochory could have evolved in the Cretaceous among a diversity of dispersal mechanisms in the absence of sophisticated animal dispersers. Its rarity today can be accounted for by the major opportunity cost that could make it too expensive to maintain as a strategy that is only energetically justified when alternative seed dispersal mechanisms are limited.

Although ballochory selects for seed traits that conflict with those optimal for secondary dispersal by such long‐distance vectors, the lack of an energetic trade‐off for investment in ballochory or myrmecochory (Chen et al. 2019) likely explains the widespread prevalence of this two‐stage combination among ballistic plants, with Chen et al. (2019) reporting 78 records of diplochory among 148 species exhibiting either myrmecochory or ballochory. While secondary dispersal by ants can mitigate against it, there is clearly a major opportunity cost involved in adapting mechanisms for dispersal that preclude the chance for long‐distance transport, so it is perhaps no surprise that it is an uncommon form of seed dispersal.

## 5 Discussion

While ballistic dispersal has been well‐studied from a mechanical point of view, until now it has been subject to little review of its evolutionary ecology. As has been discussed, ballistic dispersal is a strategy that allows plants to disperse high proportions of their seeds beyond the parental canopy (Narbona et al. 2005; Beaumont et al. 2009; Rickert and Fracchia 2010) even with relatively short dispersal distances, typically below 5 m (Vittoz and Engler 2007). This movement facilitates escape from distance‐ and density‐dependent mortalities, as predicted by the escape hypothesis for seed dispersal. Based on current evidence, it seems plausible that predation pressure on the parent plant and/or on the ground beneath can potentially contribute to the evolution of ballochory, but the ability of the strategy to facilitate escape from competition from conspecifics or increase recruitment of secondary ant dispersers represents alternative or additional selective forces that also encourage its evolution. To counter several selective advantages, the high opportunity cost of ballochory appears to account for its relative rarity among extant plants.

Future consideration of the triggers of explosive seed release could provide valuable insights in further consideration of the selective benefits of ballochory. For instance, non‐autonomous release of seeds in response to physical disturbance could incur a fitness cost when immature seeds are dispersed. *Cardamine hirsuta* seeds are released upon physical disturbance to seed capsules, which could function to escape herbivores (Vaughn et al. 2011). Similarly, *Cardamine scutata* seed capsules appear to burst as an inducible physical defence to predatory attack by caterpillars (Yano 1997). However, as well as physical disturbance from seed predators, there are other factors that could influence this non‐autonomous release: wind, pressure from desiccation, movement of neighbouring plants or seed pods, and movement of non‐predatory animals. Whatever the trigger, we suggest that consideration should be given to these cases of non‐autonomous release since they appear to represent another potential cost to the strategy. If seeds are released prior to maturity, they may have reduced germination success due to less maternal investment, and they may experience limited dispersal due to incomplete seed growth that results in sub‐optimal ballistic release. We suggest future research into the effects of seed maturity on the success of seedlings to scrutinise this line of enquiry. As a starting point, the magnitude of physical stimuli required to initiate ballistic dispersal could be investigated, and the fitness effects to immature seeds could be quantified. The triggers could be investigated experimentally by tracking seed dispersal in the lab with substrate of a contrasting colour to seeds, as in Stamp (1989) and Kaproth et al. (2023), and attempting to stimulate dispersal with various mechanical triggers (such as water or potential seed predators) and by focussing motion‐sensitive cameras on ballistic plants. In terms of fitness costs, trials could be designed that compare the germination success of seeds dispersed following physical disturbance to seeds dispersed naturally by ballochory.

In the absence of specific studies on the triggers of ballistic dispersal, understanding the evolution and maintenance of this dispersal trait is impeded. Since water is involved (either through absorption or evaporation) in the process of explosive pod dehiscence, it appears that future research should interrogate the influence of weather variables on the timing of ballistic release. If future research corroborates evidence that plants can exert autonomous control over the timing of seed release when triggered by the touch of a potential predator (Yano 1997) or by endogenous heat production (deBruyn et al. 2015), the hypothesis presented here that ballochory functions as a predator escape strategy will be advanced.

Investigating the effect of ballistic dispersal on density‐dependent predation would also help to forward understanding of ballochory's evolution. Following up Ohkawara and Higashi's (1994) experiments with two further treatments, a true ballistic seed dispersal kernel and a control distribution of seeds dropped passively from parent plants, would be insightful and represent more realistic densities than the evenly spaced treatments they adopted. In this follow‐up experiment, it would be beneficial to use a remote video feed to observe seed predators and minimise any influence of human presence on predator behaviour.

Although past efforts have identified no ecological correlates of ballochory, investigating correlations between environments where seed predation is expected to be high, such as arid environments (Morton 1985), and the frequency of ballistic dispersal would also be valuable. In addition, considering ballochory as a predator escape strategy raises questions about a potential link to other antipredator strategies. For example, can patterns between ballochory and herbivore defence chemicals be identified within clades, and if so, which trait comes first, or does ballochory lessen the selection pressure for defensive chemicals? Similarly, taking plant phylogeny into account would prove valuable in guiding understanding of ballochory's relationship to secondary myrmecochory. For instance, the question of whether elaiosomes evolved before or after ballistic dispersal could be addressed in addition to quantifying how many of ballochory's independent evolutions are associated with elaiosomes.

## 6 Conclusion

Here, we consider the available evidence to promote the hypothesis that selection for ballochory may have been driven (at least in part) by predation pressure on and underneath parent plants, but we should also consider how alternative forces of negative density‐dependent kin competition and directed dispersal to advantageous sites for establishment could contribute to the selective force that drives the evolution of explosive seed release. Perhaps ballochorous species need to be considered on a case‐by‐case basis to discern whether ballochory can be produced by different selective forces, since the evidence considered demonstrates multiple adaptive benefits. The unique ability of ballochory as an autochorous strategy to guarantee (and perhaps control the timing of) seed dispersal represents an advantage over strategies that make use of uncertain external vectors, and the short dispersal distances offered likely reflect a trade‐off between escaping natural enemies and being transported to suitable sites for establishment in patchy environments. The major opposing force to the evolution of ballochory is the preclusion of long‐distance seed transport due to morphological constraints on seeds. We have identified several lines of enquiry to allow the scrutinization of the ideas offered, and thus encourage a shift in the focus of ballistic dispersal research toward the strategy's evolutionary ecology.

## Author Contributions

**C. R. Sharpe:** conceptualization (supporting), investigation (equal), writing – original draft (lead). **G. D. Ruxton:** conceptualization (lead), investigation (equal), supervision (lead), writing – original draft (supporting), writing – review and editing (lead).

## Conflicts of Interest

The authors declare no conflicts of interest.

## Data Availability

Data sharing not applicable.
